# Morphological Fabrication of Equilibrium and Auditory Sensors through Electrolytic Polymerization on Hybrid Fluid Rubber (HF Rubber) for Smart Materials of Robotics

**DOI:** 10.3390/s22145447

**Published:** 2022-07-21

**Authors:** Kunio Shimada

**Affiliations:** Faculty of Symbiotic Systems Sciences, Fukushima University, 1 Kanayagawa, Fukushima 960-1296, Japan; shimadakun@sss.fukushima-u.ac.jp; Tel.: +81-24-548-5214

**Keywords:** auditory sensor, equilibrium sensor, cutaneous receptors, mimesis, rubber, electrolytic polymerization, hybrid fluid (HF), cochlea, saccule, robotics, smart material

## Abstract

The development of auditory sensors and systems is essential in smart materials of robotics and is placed at the strategic category of mutual communication between humans and robots. We designed prototypes of the rubber-made equilibrium and auditory sensors, mimicking hair cells in the saccule and the cochlea at the vestibule of the human ear by utilizing our previously proposed technique of electrolytic polymerization on the hybrid fluid rubber (HF rubber). The fabricated artificial hair cells embedded with mimicked free nerve endings and Pacinian corpuscles, which are well-known receptors in the human skin and have already been elucidated effective in the previous study, have the intelligence of equilibrium and auditory sensing. Moreover, they have a voltage that is generated from built-in electricity caused by the ionized particles and molecules in the HF rubber due to piezoelectricity. We verified the equilibrium and auditory characteristics by measuring the changes in voltage with inclination, vibration over a wide frequency range, and sound waves. We elucidated experimentally that the intelligence has optimum morphological conditions. This work has the possibility of advancing the novel technology of state-of-the-art social robotics.

## 1. Introduction

For the development of the behavioral interactions between humans and robots in the fields of, for example, nursery and communication, human-like intelligence, such as the five senses, is crucial. This helps realize supplemental aiding by a robot in our social life. In particular, auditory, olfactory, and tactile sensing is required in the integrated systems of electro-mechanical structures installed with variegated sensors. Unlike conventional rigid materials that are embedded or installed in ordinary robots (e.g., metals and plastics), soft materials facilitate the requirements as human-like flexible and elastic components. These materials are considered to belong in the field of smart material or soft robotics [1] and have been developed as the soft tactile skin that mimic the human senses of robots, such as artificial skin [2] and auditory materials and systems [3]. The artificial tactile skin is currently demonstrated as electronic skin (i.e., e-skin) [4,5,6,7,8], which is integrated with elastomeric substates and sensibility-induced fillers, and whose structure is assembled through configuration using a predominantly chemical process. Therefore, the production process of e-skin is generally complicated in many cases. Other propositions of structuring the artificial tactile skin use the same process wherein elastomers and fillers are utilized [9,10]. Meanwhile, auditory soft materials and systems are simulated by biologically and anatomically mimicking the human ear. These systems include inner ear, hair cell, cochlea, and semicircular canals. Optical nerve systems mimic the human eye. The perception realized using these mimicked artificial organs is the same as that of tactile skin. This is because their perception is based on the physical principle that they are reacted with, for example, by causing a movement to generate a voltage. In particular, this voltage is induced by either piezoresistivity or piezoelectricity [11]. In the former case, the voltage is generated within the artificial organs after certain voltage is externally applied to these organs using a power source. In the latter case, the voltage is induced by ionic behavior within artificial organs without requiring a power source; therefore, the advantage of these systems is that they can function without a power supply unit. Therefore, it is convenient and effective to utilize the principle of perception and the production process of tactile skin to realize auditory soft materials and systems [12,13,14,15]. Shimada proposed and continues to investigate the rubber-made soft and highly expansible haptic artificial skin, called magnetic compound fluid rubber (hereinafter, MCF rubber) [16] or simply hybrid skin (hereinafter, H-skin) [17], and mechano-thermo receptors simulating human cutaneous receptors [18]. The essential viewpoints of proven artificial auditory cutaneous receptors are the use of a piezo-resistor to induce voltage or electric current through the deformation of the material and the use of soft rubber. HF rubber for hair cells fulfills these essential requirements.

MCF is a magnetically responsive intelligent colloidal fluid that is formed using a 1 μm-thick layer of ordered metal particles (e.g., Ni, Fe, and Cu) and a 10 nm-thick layer of ordered sphere magnetite (Fe_3_O_4_) particles, and it is further coated by oleic acid surfactant in a solvent (e.g., water, kerosene, and silicone oil). By application of a magnetic field, many needle-like magnetic clusters are created in MCF to enhance its electrical and thermal conductivity. Therefore, when MCF is compounded in a rubber, such as natural rubber (NR) and chloroprene rubber (CR), it becomes conductive rubber. Furthermore, MCF rubber can be solidified through electrolytic polymerization, making the vulcanization process using sulfur unnecessary. The electrolytic polymerization process requires the solvent to be water soluble. Because NR and CR are water-soluble diene rubbers, they can be electrolytically polymerized. For non-diene rubbers (e.g., silicone rubber (Q)), which are soluble in kerosene, because diene and non-diene rubbers combine well when mixed with polyvinyl alcohol (PVA), Q can be electrolytically polymerized by mixing it with either NR or CR and PVA. Currently, soft materials for obtaining thin or flexible sheets, such as e-skin, are generally produced using polydimethylsiloxane (PDMS) the same as Q because it is tractable and is a major commercial material in molding. Therefore, the electrolytic polymerization method used for Q is essential to make rubber-based sensory systems for robotics functional. In addition, electrolytic polymerization can be achieved by replacing Q with urethane rubber (U). The surfactant and Fe_3_O_4_ powder can be mixed, instead of MF. In particular, the magnetically responsive intelligent colloidal fluid that is compounded by metal and Fe_3_O_4_ particles, surfactant, and PVA in a solvent that is mixed with water, kerosene, and silicone oil is called hybrid fluid (HF) [19]. Due to the presence of PVA, HF is soluble in both diene and non-diene rubbers. Thus, this soluble rubber is called HF rubber [19]. By using HF rubber, we can produce a soft and stretchable mimetic organ of perceptual cells in the human body. This includes free nerve endings, Meissner corpuscles, Pacinian corpuscles, Krause and bulbs, and Ruffini endings [18]. These receptors have variegated intelligence with a discrete structure so that the morphologically mimicry to produce their configuration is difficult. Few studies have addressed the fabrication of these individual styles. Therefore, our proposed morphological fabricate that mimicked these receptors is novel. We can also fabricate artificial U-rubber skin embedded with artificial cutaneous receptors to mimic human skin tissue. The five cutaneous receptors are suitable for use in our proposed study. Although we can utilize any of these receptors, considering the page length restrictions, we selected free nerve endings and Pacinian corpuscles for this study. The other receptors will be demonstrated in future studies.

The production of rubber-based soft materials using HF rubber requires fewer procedural steps because the techniques of electrolytic polymerization are utilized. Therefore, we adopted the HF rubber-based production techniques in the present study. Furthermore, it is important that the present production technique is conducted on with utilizing materials such as HF rubber different from conventional materials in other previous researches because the production of the smart materials of robotics, such as equilibrium and auditory cutaneous receptors just made of rubber, is novel. The electrolytic polymerization enables not only the solidification of rubber, as described in latter sections, but also adhesion between the metal wire, which is used as an electrode, and rubber [20]. In this manner, we demonstrate the possibility of the fabrication of novel rubber-based auditory soft materials and systems using the electrolytic polymerization technique. To achieve this, we investigated the characteristics of the HF rubber-made equilibrium and auditory cutaneous receptors and showed that they mimic those of the hair cells in the utricle and saccule at the vestibule of the human ear.

## 2. Anatomical Background

The present artificial equilibrium and auditory receptors can be mimicked by the human auditory organ of the inner ear.

**Figure 1:**
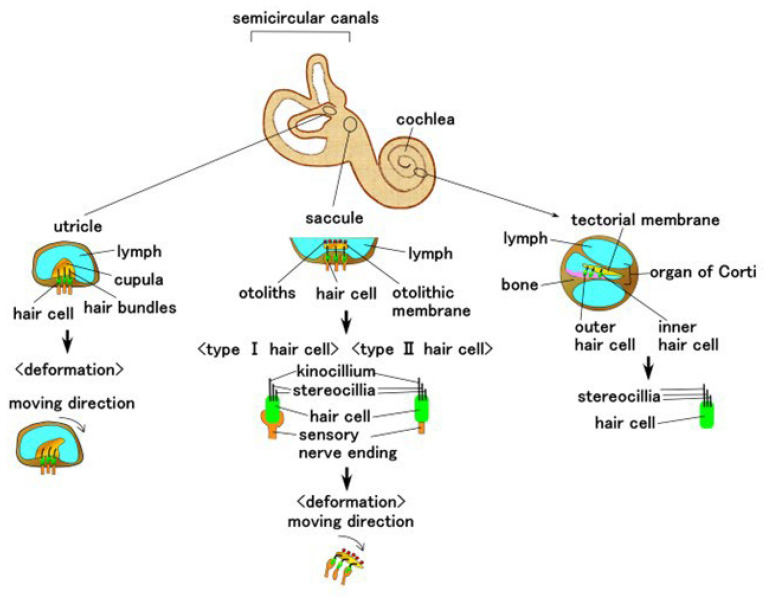
Schematic anatomical diagram showing the general structure and position of semicircular canals and cochlea in the human ear.

Living organisms (e.g., animals and fish) have mechano-sensing for detection and transmission of vital information through stimuli. Their sensing capabilities are morphologically adapted to natural circumstances, and the typical configuration for achieving this sensitivity is demonstrated using a hair cell or cupula that mimics the same human auditory organ [21,22,23,24,25,26,27,28,29]. The human auditory organ of the inner ear is shown in Figure 1.

The hair cell has multiple thin hairs in three regions: the utricle, the saccule, the and organ of Corti in the cochlea. The utricle is immersed in a jelly-like cap, known as the cupula, and is activated by the deformation of the cupula according to the stimuli from all directions. The saccule is reacted by the deformation generated by the movement of the otolithic membrane induced by the otoliths mounted on the otolithic membrane; the utricle and saccule are related to the sensitivity of equilibrium. The organ of Corti in the cochlea is responsive to acoustic sensitivity. The sensitivity of movement, equilibrium, and acoustics can be realized by the artificially mimicked configuration of hair cell or cupula and polymer or hydrogel. The artificial hair cell with cantilever beam-like configuration with multiple piezoelectric layers has been demonstrated for the motion situation under acceleration [22,30] and for the acoustics in the cochlea [31]. Meanwhile, the hair cell has a membrane-type configuration that contains a piezoelectric layer and multiple electrodes at the cochlea for sound [32,33,34]. The other case of the hair cell is demonstrated using magnetic switch systems with magnetic probe and magnetic nanoparticles to realize a magnetically induced motion [26,35,36]. Artificial cupula designed using PDMS with several thin polyvinylidene fluoride nanofibers placed on a piezo-resistor base has been proposed to demonstrate sound-induced movement [21,23,24,25,28]. An artificial three-dimensional (3D) printed ear with miniaturized coiled antenna has been proposed for sound detection and recognition [37]. To achieve equilibrium, artificial semicircular canals, which had hair cell bundles immersed in a cupula made from vertical graphene nanosheets and PDMS, and which were realized by utilizing microelectromechanical systems (MEMS), have been proposed [38,39,40]. Meanwhile, other artificial semicircular canals have been used to demonstrate the interaction between the outer magnet and inner spherical ferrofluid body placed in a loop [41].

As shown in Figure 1, the hair cell is constructed using thin and elastic hair-like rods and receptors located at the sensory nerve ending. To enhance the sensitivity of its deformation by movement, the hair cell is placed inside a soft material, such as cupula, or the otoliths are placed on a soft material, such as saccule.

## 3. Materials of Mimicked Receptors

**Figure 2:**
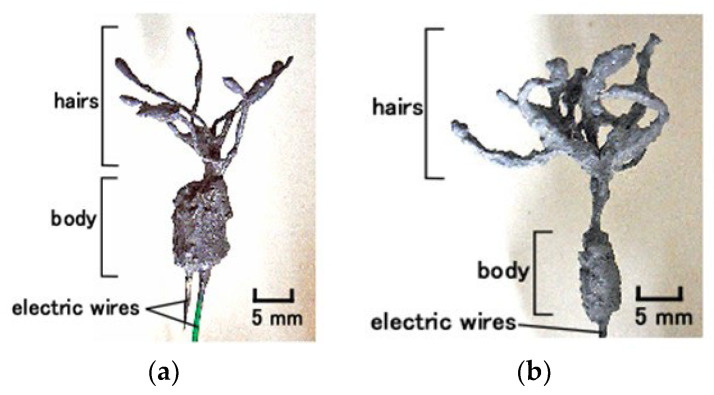
Images of the designed cutaneous receptor fabric produced using electrolytic polymerization of HF rubber: (**a**) free nerve endings and (**b**) Pacinian corpuscles.

This study deals with two types of hair cells adhered to otolithic membrane, such as saccule, which is used for equilibrium sensing, and the bare hair cell surrounding a lymph, such as cochlea, which is required for sound sensing. The hair cells mimicking saccule and cochlea are formed using two structures: thin and elastic hair-like rods and receptors at sensory nerve endings (Figure 1). The first structure is similar to that of hair bundles present in the utricle, kinocilium or stereocilia, saccule, and cochlea. This structure, in the case of the cochlea, is sensitive to sound waves. The second structure is sensible to the motion of the former structure, which is induced by the deformation of the cupula in the case of the utricle and by the motion of otoliths and otolithic membrane in the case of saccule. To mimic both these structures, in this study, we utilized two types of free nerve endings (Figure 2a) and Pacinian corpuscles (Figure 2b). The structure of free nerve endings is layered type, whereas that of the Pacinian corpuscles is cylindrical. As shown in the configuration, their design has two parts: hairs, which are affected by external motion or sound wave, and a body, which is responsible as the kinetic and audible sensory receptor.

Utilizing the cells responsible for perception in the human cutaneous tissue, nerve endings of the receptor, Meissner corpuscles, Pacinian corpuscles, Ruffini endings, etc., in the existing kinetic and auditory sensor is highly effective because the structure of this cutaneous receptor is similar to that of the receptors in the auris interna. As supporting evidence, artificial mechanoreceptors mimicking tactile receptors have been verified to be applicable as audio receptors [12,13,14,15].

The components of HF are 3 g water, 3 g kerosene, 3 g silicon oil (KF96 with 1-cSt viscosity, which would solidify Q with PDMS; Shin-Etsu Chemical Co., Ltd., Tokyo, Japan), 21 g PVA, 3 g Fe_3_O_4_ particle (Fujifilm Wako Chemicals Co., Ltd., Osaka, Japan), 3 g Fe particle (M300, about 50-μm particles; Kyowa Pure Chemical Co., Ltd., Tokyo, Japan), and 4 g sodium hexadecyl sulfate aqueous solution. All ingredients are mixed together by an agitator with slow speed under evacuation by a vacuum pump for removing air bubbles [19]. After mixing rubber latex and other ingredients with the HF using a super-sonic vibrator, as specified in Table 1, HF rubbers 1-4 were electrolytically polymerized. We considered carbonyl Ni powder, which has particle sizes in the order of microns, with bumps on the surface.

HF rubber 1 is for fabricating the hair cell by the rubber, HF rubber 2 for insulator as condenser whose configuration is similar to electrolytic capacitor, HF rubber 3 for outer cover of the condenser structured with HF rubber 2, and HF rubber 4 for adhesive, which has role of adhesion between rubbers, and between rubber and electric wires.

In preliminary phase, HF rubber 2 was solidified by electrolytic polymerization twice under 180-mT magnetic field, which was achieved using permanent magnets set on the outer side of the electrodes while ensuring a 0.5 mm distance between electrodes. This procedure lasted for 1 min. In case the solidified HF rubber 2 was required to be thicker, we used a 1 mm distance between electrodes, and electrolytic polymerization was performed for 2 min. HF rubber 2 is porous, which allowed glycerin infiltration with vacuum defoaming. This process lasted for 2 h. As a result of using glycerin, HF rubber 2 became dielectric. On the other hand, HF rubber 3 is solidified by electrolytic polymerization once under 180 mT magnetic field while maintaining a distance of 0.5 mm between electrodes, and the process lasted for 1 min. In case the solidified HF rubber 3 was required to be thicker, we used 1 mm electrodes distance, and the electrolytic polymerization duration lasted for 5 min. HF rubber 3 prevents aridity, which allows it to be used as the outer cover.

The next phases of fabricating the hair cell after preparing HF rubbers 1–4 was demonstrated in the previous study [18]. These phases, related to free nerve endings, are summarized as follows.

**Figure 3:**
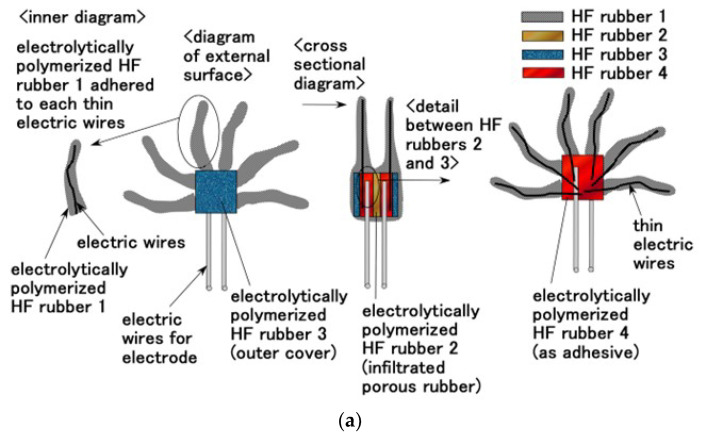

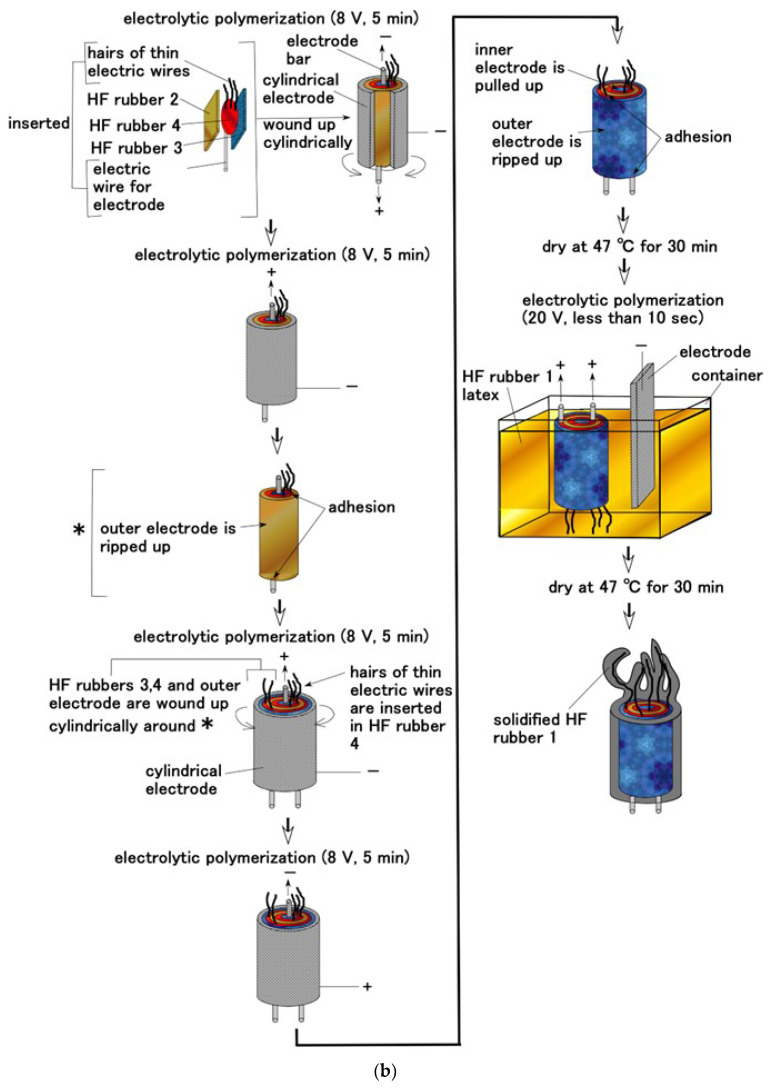
Schematic diagram showing the production and fabrication procedures of HF rubber receptors: (**a**) free nerve endings and (**b**) Pacinian corpuscles.

In the first phase of combining HF rubbers (Figure 3a), several extremely thin electric wires, approximately 0.1 mm in diameter; electric wires of sensor electrodes with an outer diameter of approximately 0.8 mm; and seven thin silver-gilt electric wires of approximately 0.1 mm in diameter were inserted between HF rubbers 2 and 3 to be electrolytically polymerized with HF rubber 4 latex for 5 min without a magnetic field. HF rubber 4 is solidified by electrolytic polymerization so that the electric wires can be bonded to the rubber; therefore, HF rubber 4 served as an adhesive. Here, it should be noted that the side of HF rubber 3 was in contact with HF rubber 4. The exposed side of HF rubber 3 facing outward was the surface on the cathode side during electrolytic polymerization at the preliminary phase; it had an uneven shape. If the surface is on the anode side during electrolytic polymerization, it will not be able to adhere well to U; in which case, the produced hair cell must be immersed afterward. Owing to its concave and convex shape, the surface that was on the cathode side adhered to U. In addition, HF rubber 2 was sandwiched between them and electrolytically polymerized with HF rubber 4 latex for 5 min without a magnetic field.

In the next phase, the hair cell obtained from above process was immersed in HF rubber 1 latex and electrolytically polymerized for less than 10 s while ensuring that the electric wires for electrode with 0.8 mm outer diameter was the anode and that the cathode was set apart from the produced hair cell without a magnetic field. Consequently, HF rubber 1 adhered to the several extremely thin electric wires that were 0.1 mm in diameter. HF rubber 1 has role of soft and elastic cover of the thin electric wires. The electrolytic polymerization duration requires shorter lapse so that the production is useful and convenient and requires less time. The hair cell thus fabricated consists first of hairs, in which thin electric wires 0.1 mm in diameter are immersed, and then body, in which HF rubbers 2 and 3 are sandwiched (Figure 2a).

## 4. Methods of Mimicry

The fabrication process of Pacinian corpuscles is shown in Figure 3b. The previous study [18] demonstrated Pacinian corpuscles with a layered structure; however, that with a cylindrical structure has not been proposed yet. The electrolytic polymerization process in this case is the same as that used for the layered type. However, the configuration at the electrolytic polymerization is different; the shape of the electrodes at the electrolytic polymerization was changed to cylindrical by winding up the electrode plate.

For both hair cells, when several extremely thin electric wires with 0.1 mm in diameter were attached to the electric wires for electrodes that have 0.8 mm outer diameter, the extremely thin wires became conductive. Thus, we could choose whether these extremely thin wires should be conductive.

**Figure 4:**
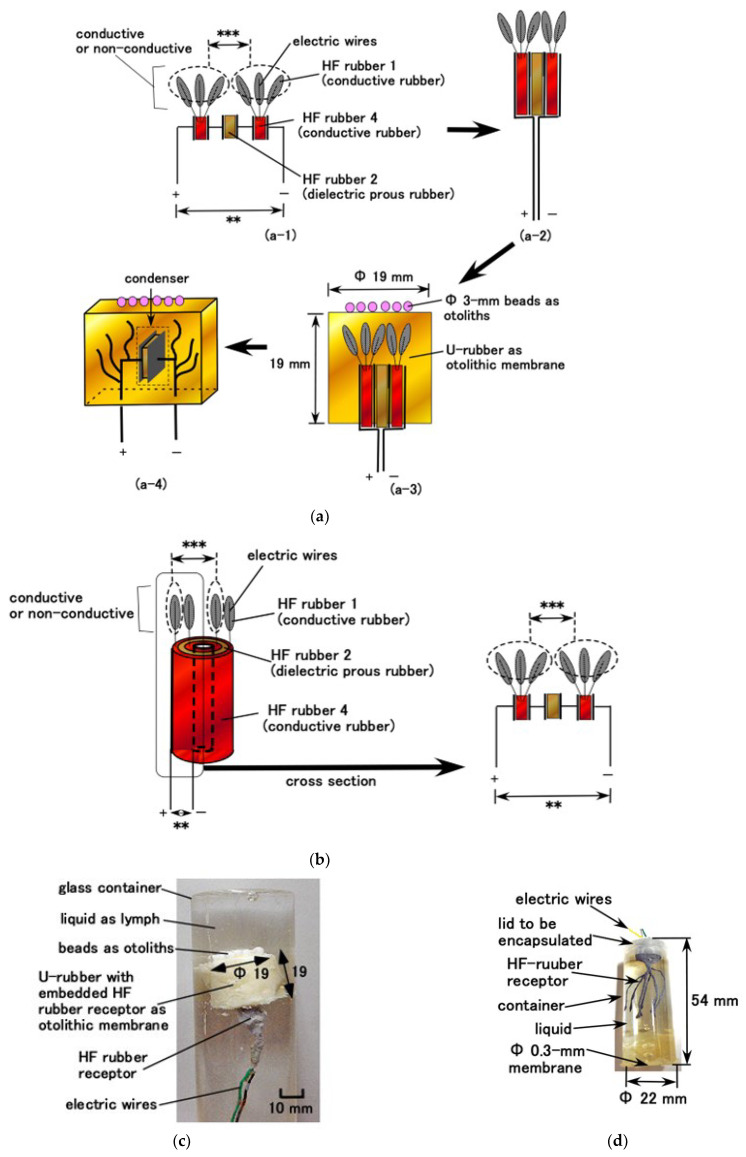

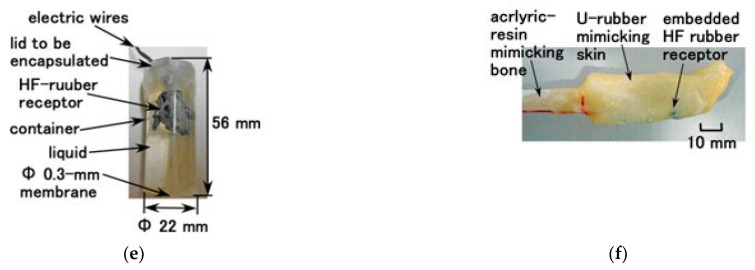
Schematic diagrams and images of fabricated receptors and sensors produced by electrolytic polymerization of HF rubber: (**a**) equivalent electric circuit of free nerve endings; (**b**) equivalent electric circuit of Pacinian corpuscles; (**c**) equilibrium sensor mimicking a saccule; (**d**) auditory sensor with free nerve endings mimicking the organ of Corti; (**e**) auditory sensor with Pacinian corpuscles mimicking the organ of Corti; (**f**) tactile receptors embedded in U-rubber mimicking a finger.

Through electrolytic polymerization of HF rubber, the particles of Fe_3_O_4_, Fe, Ni, and TiO_2_ and the molecules of the rubber and water were ionized such that HF rubber has p-type and n-type semiconductor-like roles in the form of A^−^ (A is the acceptor) and D^+^ (D is the donor) ions. The induced electrons and holes were mobile; due to which, they cancelled each other electrically in the adjacent area between them, leading to the formation of a depletion layer. Meanwhile, the ionized particles and molecules were in a static state so that the built-in voltage and current were caused by piezoelectricity; this behavior is similar to that of a condenser. The equivalent electric circuit of the hair cell shown in Figure 3a,b are shown in Figure 4a,b, corresponding to free nerve endings and Pacinian corpuscles, respectively. As mentioned previously, the difference between these two hair cell types is the layered flat type and cylindrical configuration. The built-in voltage is evaluated as *A* in the figure. If several extremely thin electric wires with 0.1 mm diameter and the electric wires for electrode with 0.8 mm outer diameter are brought in contact, conductive current or dielectric voltage can be generated between the thin electric wires, as denoted by ***, in Figure 4a,b. Then, the built-in voltage can be evaluated by measuring **. Regarding layered flat type, the measured built-in voltage is the voltage generated in the condenser, as shown in a-4 in Figure 4a. By the deformation of U-rubber induced by the outer force or the own motion, not only the condenser but the bilateral electric circuits of the condenser are deformed. Therefore, the built-in voltage changes. On the other hand, regarding cylindrical type, the configuration can be considered the same one of layered flat type: the configuration from the right figure in Figure 4b is the same one as shown by figures from a-2 to a-4 in Figure 4a.

**Figure 5:**
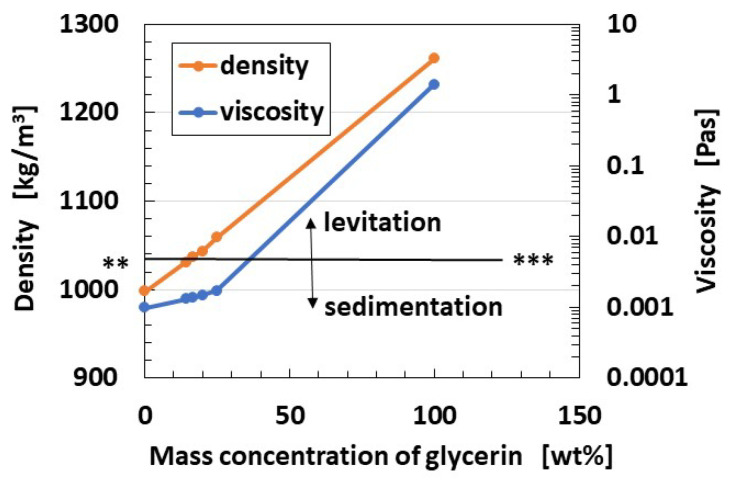
Density and viscosity of the liquid used in the equilibrium sensor mimicking a saccule.

**Figure 6:**
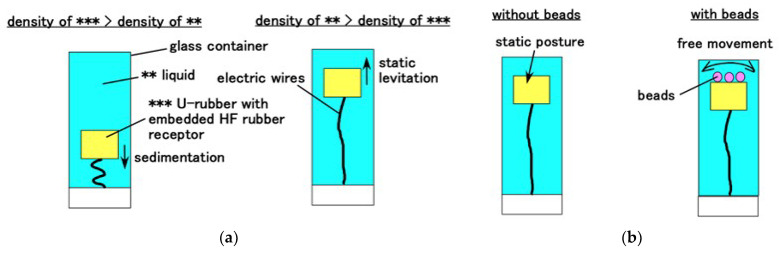
Posture of the hair cell in the equilibrium sensor mimicking a saccule: (**a**) relation between the levitation and density of the liquid and (**b**) effect of beads mimicking otoliths on the posture.

For the hair cell adhering to the otolithic membrane, such as saccule, where it is used for equilibrium sensing, the fabricated HF rubber hair cell is molded in cylindrical U-rubber (Human skin gel, 0-solidity; Exseal Co., Ltd., Gihu, Japan), which simulates the otolithic membrane and many Al_2_O_3_ beads with 3 mm diameter (C.C., HD ball; Nikkato Co. Ltd., Osaka, Japan), which are used to simulate otoliths, are put on the U-rubber, as shown in Figure 4a. Subsequently, the fabrication of the hair cell mimicking a saccule is performed (Figure 4c). Because in human ear, the otolithic membrane is immersed in lymph at saccule, the fabricated hair cell is immersed in a container filled with a liquid, which is a mixture of glycerin and water, and its density is high enough that it caused the U-rubber to levitate. The limit line to be levitated is shown as ** and *** line in Figure 5, which satisfies the condition that the proportion of water is less than five times that of glycerin, and the density of the mixture is 1.037 × 10^3^ kg/m^3^. The present specimen used the five times proportion to levitate the mimicked hair cell, as shown in Figure 6a. Furthermore, the cumulative weight of Al_2_O_3_ beads is also important. Each Al_2_O_3_ bead had density of 3.6 × 10^3^ kg/m^3^ and weighed 0.0472 g. To prevent the sedimentation of the mimicked hair cell, 16 Al_2_O_3_ beads with a total weight of 0.7552 g were found to be optimal solution that allowed the mimicked hair cell to move laterally, as shown in Figure 6b. When the number of Al_2_O_3_ beads was higher than 16, the mimicked hair cell sedimented or broke down. However, when the number of Al_2_O_3_ beads was less than 16, the lateral fluctuation of the mimicked hair cell was smaller.

For the hair cell, such as cochlea, which is used for sound wave sensing, the fabricated HF rubber hair cell is enclosed in a container, as shown in Figure 4d,e in the case of free nerve endings and Pacinian corpuscles, respectively. The liquid used was either glycerin or water. The cylindrical container made of acrylic resin had an inner diameter of 19 mm. At one end of the container, a 0.3 mm-thick soft membrane made of Q-rubber was adhered. The components of this membrane consisted of silicon oil (KE1300T, Shin-Etsu Chemical Co., Ltd., Tokyo, Japan) and thinner in the ratio of 3:1. Young’s modulus is 0.922 GPa measured by using a tensile and compressive machine (SL-6002; IMADA-SS Co., Ltd., Toyohasi, Japan). Thus, this configuration is easy to install in a robot as a part of sensory receptor, for example, in an artificial ear.

As another sensory configuration, we can demonstrate artificial tactile human hand finger using a sensory receptor. This has been presented in a previous study [18]. As shown in Figure 4f, the HF rubber hair was embedded in U-rubber and molded as a finger. This case was used to be compared in terms of sensory characteristics with saccule and cochlea fabricated using HF-rubber.

## 5. Principle of HF Rubber Receptor

**Figure 7:**
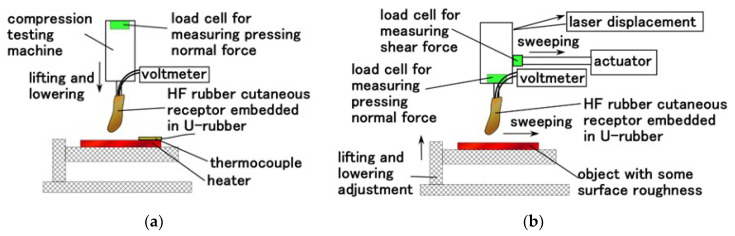
Schematic diagram of the experimental apparatus for measuring tactile and thermal senses: (**a**) applying a normal force (NFE) and (**b**) applying a shear force (SFE).

We confirmed the effectiveness of the sensory receptor by measuring its tactile sensing capabilities using normal and shear force. The former was implemented using the normal force experiment (NFE) apparatus used in our previous study, whereas the latter used a shear force experiment (SFE) apparatus [16]. As for NFE, the finger is moved to touch a rigid flat plate or heater. The up and down motion was repeated five times using a tensile and compressive machine (SL-6002; IMADA-SS Co., Ltd., Toyohasi, Japan) at a velocity of 100 mm/min for a rigid plate and 300 mm/min for a heater, as shown in Figure 7a. As for SFE, the finger was moved to contact an object with a rough surface using an actuator with 50 mm/min sweeping speed and approximately 0.02 N normal force, as shown in Figure 7b. The normal force on the finger and the sweeping distance were measured using a load cell and laser displacement, respectively. The rubbed object is two types: an object with surface roughness was achieved using sandpaper and that with both a concave and convex body. The voltage induced in the sensor was measured using a voltmeter (PC710, Sanwa Electric Instrument Co. Ltd., Tokyo, Japan).

The free nerve endings have already been demonstrated in our previous study [18], and, similarly, Pacinian corpuscle is shown in the present study. The present Pacinian corpuscle is a cylindrical type, whereas that demonstrated in the previous study [18] is a layered plate-type.

**Figure 8:**
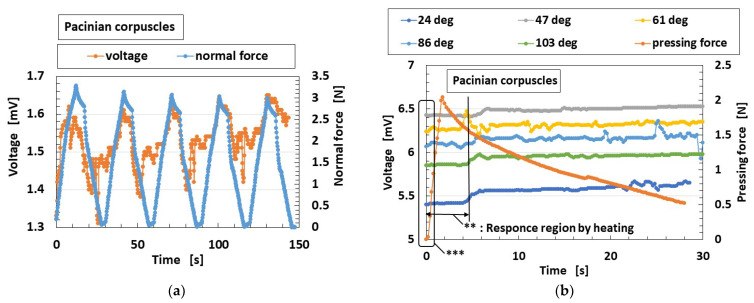

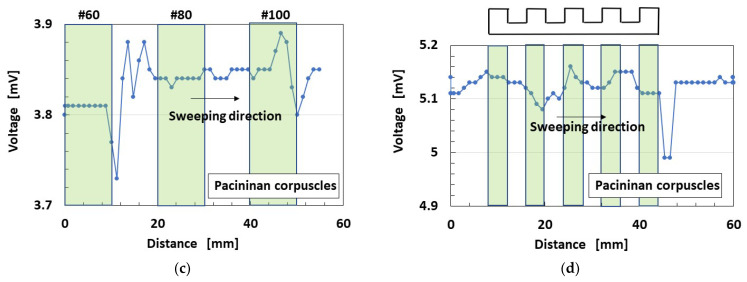
Experimental results of tactile and thermal sensing obtained in different cases: when (**a**) applying a normal force; (**b**) touching a heater; (**c**) applying a shear force on intermittently positioned sandpaper; (**d**) applying a shear force on a convex–concave-shaped body.

The HF rubber Pacinian corpuscle is sensitive to normal force, as shown in Figure 8a. This is similar to normal force sensing in the case of free nerve endings demonstrated in the previous study [18]. The Pacinian corpuscle responds to the heating body by touching vertically on the body, as demonstrated at the primary stage affected by heating as delineated ** in Figure 8b. The initial voltage as delineated *** means built-in voltage, which can easily change according to the experimental ambience. This is similar to thermal sensing in the case of free nerve endings, which is demonstrated considering the case of water-bathing compression in the previous study [18]. The voltage on the abscissa of the figure, in other words, at zero time, indicates the generated built-in voltage by the ionized particles and molecules through piezoelectricity. Considering shear force sensing, the Pacinian corpuscle is sensitive to shear force, as shown in Figure 8d, although the sensitivity is somewhat low. This is similar to shear force sensing in the case of free nerve endings, which is demonstrated in our previous study. Here, the stroked alternate sandpaper in Figure 8c has a surface roughness of #60 (*R_a_* = 30.91 μm, *R_q_* = 36.38 μm, *R_y_* = 139.4 μm), #80 (*R_a_* = 24.11 μm, *R_q_* = 28.6 μm, *R_y_* = 103.9 μm), and #100 (*R_a_* =16.01 μm, *R_q_* =19.56 μm, *R_y_* =84.5 μm). A smooth object (*R_a_* = 0.03 μm, *R_q_* = 0.03 μm, *R_y_* = 0.2 μm) was placed between the other objects of #60, #80, and #100 by introducing a sleek acrylic resin surface. The surface roughness was measured by a surface roughness measuring device (SJ-400; Mitutoyo, Co. Ltd., Kawasaki, Japan). The stroked concave and convex body in Figure 8d had a 3 mm height and a 4 mm width of gutter.

The receptor made of HF rubber is reacted with changes of built-in voltage as piezoelectricity. Therefore, when the equilibrium and auditory sensors are made with the HF rubber receptor, the receptor causes the changes to the built-in voltage by motion in the equilibrium sensor and by sound oscillation in the auditory sensor. That is a physical principle of utilizing HF rubber.

## 6. Experimental Devices

**Figure 9:**
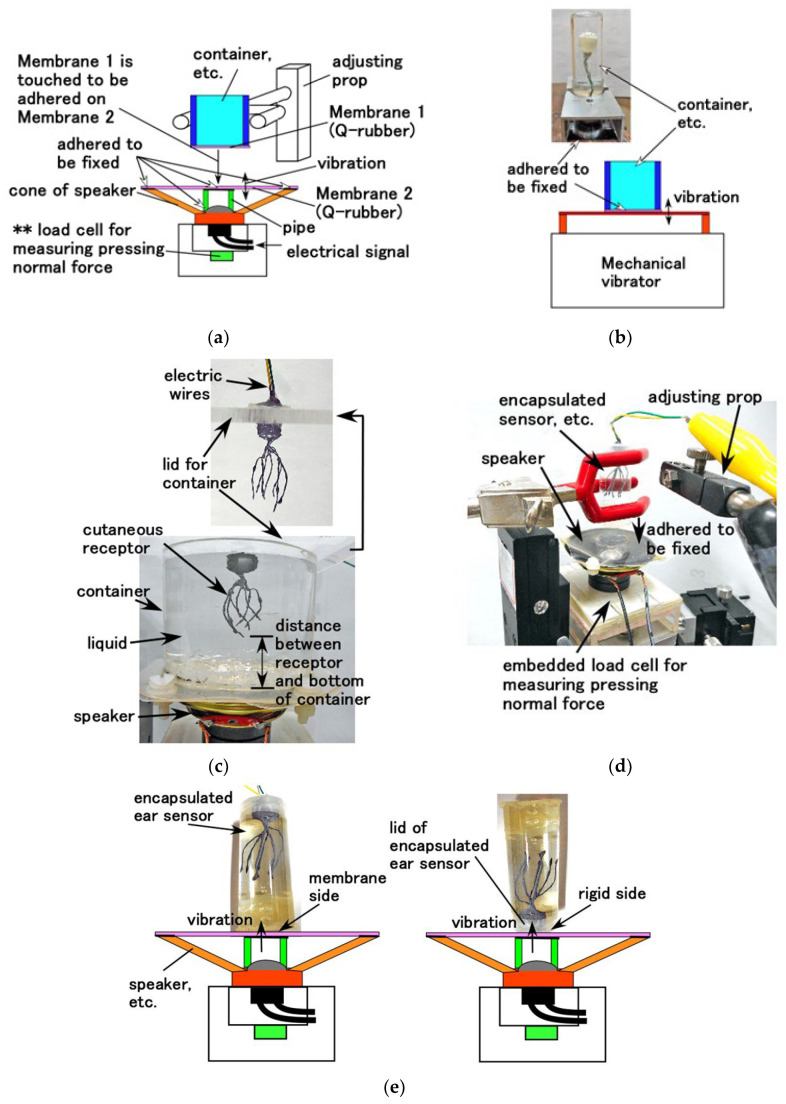
Schematic and images of the apparatus for the vibration and acoustic experiment; (**a**) vibration for a wide-ranged frequency and acoustic experiment by the speaker; (**b**) vibration by the mechanical vibrator for low frequency; (**c**) posture of the receptor in the container of the apparatus with a speaker; (**d**) posture of the sensor in the apparatus with a speaker; (**e**) posture of the auditory sensor mimicking organ of Corti in the apparatus with a speaker.

We designed the experimental setup for investigating the properties of bare and encapsuled HF rubber receptors in the presence of sound waves and dynamic equilibrium. As for the former, the performance of sound wave is relevant to the vibration. Therefore, two separate experiments were required to measure/study the vibration characteristics and the acoustic sound detection performance. Accordingly, the vibration of a speaker was used, as shown in Figure 9a. This performance is effective for measurement under high frequency range of the vibration. In contrast, for the performance of the dynamic equilibrium, mechanical vibration generated using vibration machine was also used, as shown in Figure 9b. This performance is effective for subtle measurement under low frequency range of the vibration. A rigid acrylic resin pipe was placed between the cone of a speaker and a 0.5 mm-thin membrane, labeled as Membrane 2, made of Q-rubber and silicon oil (KE1300T; Shin-Etsu Chemical Co., Ltd., Tokyo, Japan). Another 0.5 mm-thin membrane, labeled as Membrane 1, made of the same Q-rubber was adhered to the edge of an acrylic resin container and was fixed on Membrane 2. These membranes exhibited 3619 GPa Young’s modulus, which was measured using a tensile and compressive machine. The electrical signal of vibration from PC is supplied to the speaker, after which the vibration was directly transmitted to Membrane 1. The container was held by adjusting the prop, and was filled entirely by the liquid. Moreover, the HF rubber receptor was immersed in the liquid, as shown in Figure 9c. In the case of the encapsulated or bare HF rubber receptor or finger, Membrane 2 directly contacts the surface to measure the pressing normal force using the load cell **, installed at the bottom of the speaker, as shown in Figure 9d. For the encapsulated HF rubber receptor, we selected two types: one in which the side of the capsule on which Membrane 2 was fixed has thin membrane adhered to it, whereas its other side has a rigid lid. The former detects the performance of the stimuli generated in the cochlea and travelling across the eardrum, as shown in the left side image of Figure 9e. The latter detects the stimuli travelling across the bone as a rigid body involving the skull and inner ear, which is usually named as osteophony, as shown in the right-side image of Figure 9e. We considered several diameters for the container because the cross-sectional area of the cochlea depends on the sensitivity to the frequency of the sound wave. For a large cross-sectional area of inlet or outlet of the cochlea, the auditory receptor is sensitive to high frequency; whereas for a small cross-sectional area of the innermost region of the cochlea, it is sensitive to low frequency. The containers we used are labeled as Container 1 (65 mm outer diameter, 57 mm inner diameter), Container 2 (42 mm outer diameter, 38 mm inner diameter), Container 3 (33 mm outer diameter, 29 mm inner diameter), and Container 4 (22 mm outer diameter, 19 mm inner diameter); the inner diameter corresponds to the area of occupying liquid. All containers have a 50 mm height.

Furthermore, we considered mechanical vibration, as shown in Figure 9b, to investigate not only the dynamic equilibrium but also the vibration property during lower frequency range. This experiment was performed because the mechanical vibrator (Matsudaira type, UBC-4, Itoh Seiki Co. Ltd., Tokyo, Japan) can generate a vibration of less than 15 Hz, which the speaker cannot generate.

**Figure 10:**
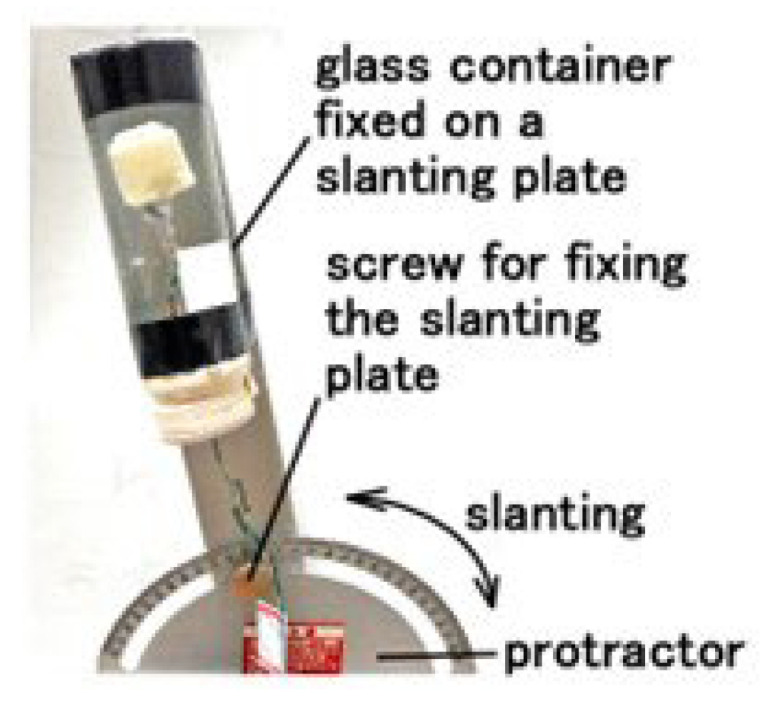
Posture of the equilibrium sensor mimicking a saccule for the slanting experiment.

To measure the equilibrium performance, the fabricated HF-rubber saccule was enclosed in a glass container, which was set up on a slanting plate whose angle is adjusted clockwise or counter-clockwise using a protractor, as shown in Figure 10. Every voltage from the sensor was measured using a voltmeter (PC710).

## 7. Equilibrium Performance

### 7.1. Methods

First, the voltage of HF-rubber saccule that was responsive to static inclination was measured while intermittently changing from the vertical position, as shown in Figure 10. By the inclination with a step of 1°, the sensor is inclined, and then it stops. The angles change in the following steps: clockwise (i.e., right slanting) up to 70°, 0° counter-clockwise, −70° (i.e., left slanting), and finally, 0°. By the inclination the U-rubber coating the receptor is deformed so that the built-in voltage changes. Care should be taken that the static state affects the changes in voltage. Next, the equilibrium receptors at dynamic motion were measured, as shown in Figure 9b. The amplitude of the mechanical vibrator is 0.64 mm in cases of horizontal and vertical vibration over low frequency ranges when using the vibrator.

### 7.2. Results and Discussion

**Figure 11:**
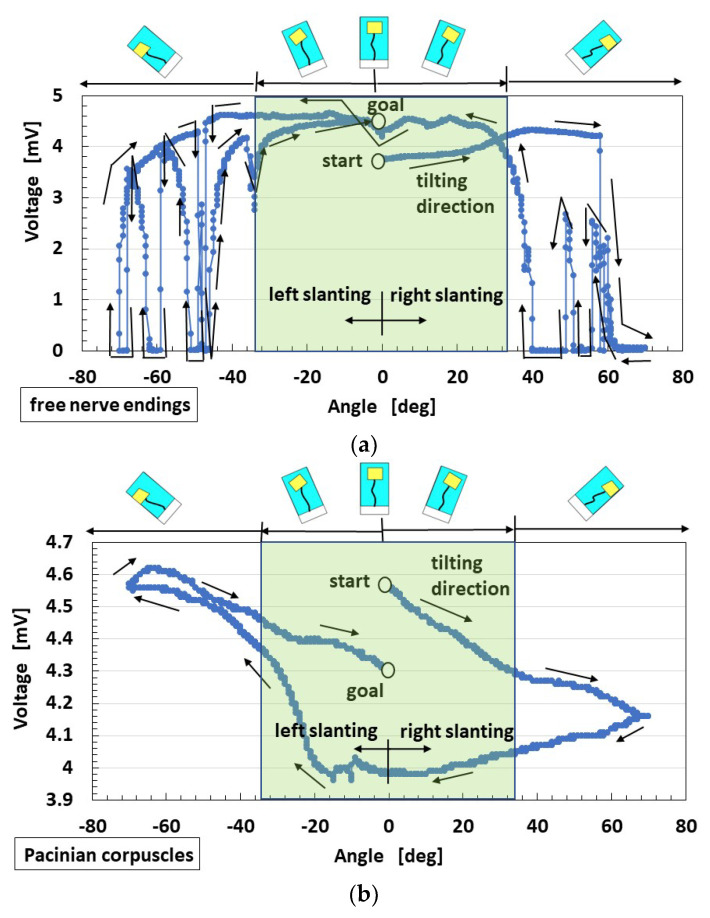
Experimental results of the slanting equilibrium sensor: (**a**) with free nerve endings and (**b**) Pacinian corpuscles.

**Figure 12:**
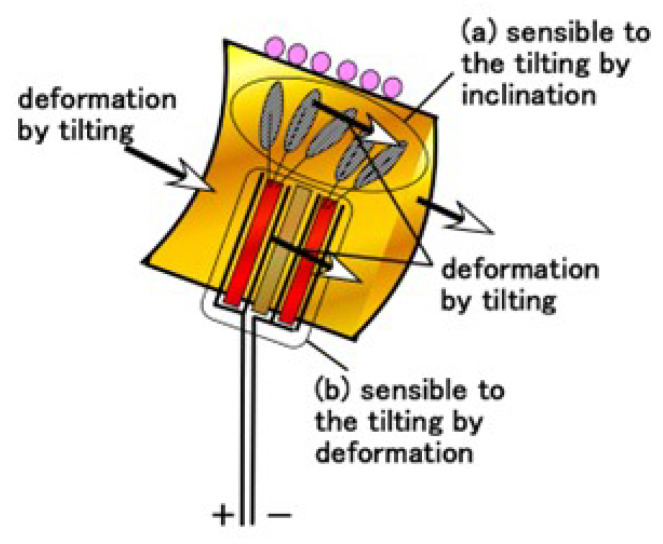
Schematic of physical model of equilibrium performance by inclination of equilibrium sensor mimicking a saccule.

The voltage of HF-rubber saccule changed from the vertical position labeled as “start”, as shown in Figure 11. The angles were finally changed up to 0°, which is labeled as “stop”, where the order direction is presented using an arrow. The angle area delineated using a pale green region in the figure provides a situation where the U-rubber does not contact the inner surface of the glass container. For the free nerve endings, in pale green area, the voltage changed gradually. Conversely, outside the pale green region, the voltage fluctuated rapidly. The cause is due to the deformation of the receptor embedded in U-rubber after the U-rubber contacts the inner surface of the glass container. The U-rubber, which has the receptor in the container, has 12.8 GPa compressive Young’s modulus, which was measured using a tensile and compressive machine. Therefore, it is elastic enough to be deformed, as shown in Figure 12. By the inclination the U-rubber coating the receptor is deformed, as shown in Figure 12, and then the voltage changes. The free nerve endings are affected by the U-rubber’s elasticity by inclination. The hair and the body of the equilibrium sensor are deformed, respectively, as delineated by *a* and *b* in Figure 12. Therefore, from Figure 4a,b, the HF rubber 2 deforms so that the dielectricity changes, and the HF rubber 4 deforms so that the conductivity changes; as a result, the built-in voltage of the sensor changes. On the other hand, the Pacinian corpuscles are not easily affected. Therefore, the qualitative defined rules between the voltage and the angle are obtainable in the case of free nerve endings and not in the case of Pacinian corpuscles. The cause is guessed to be due to the difference of the structure between layered and cylindrical configurations.

**Figure 13:**
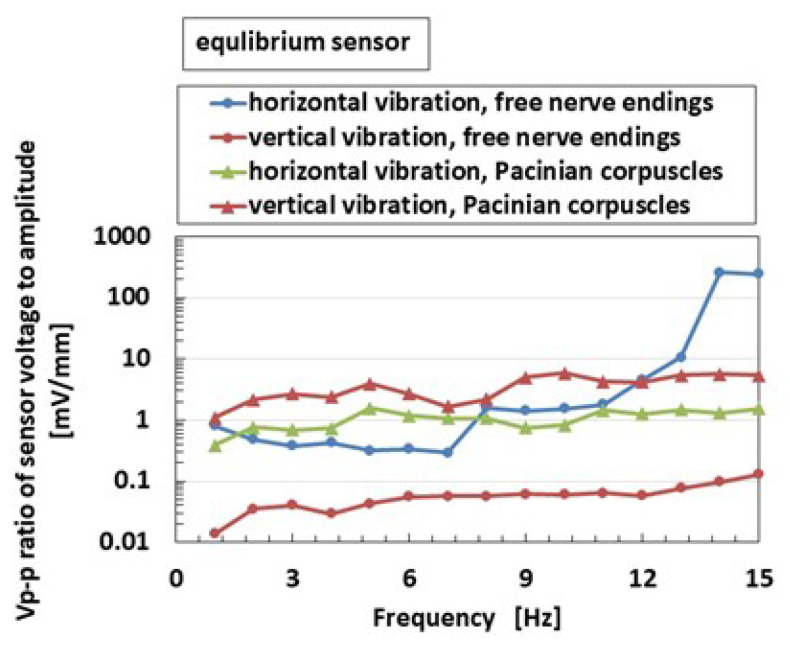
Experimental results of the vibrating equilibrium sensor by a mechanical vibrator.

The above results were obtained for equilibrium receptor at static posture. Next, equilibrium receptors at dynamic motion are shown in Figure 13. The figure maps the ratio of peak-to-peak voltage *V_p-p_* of the sensor and that of the amplitude of the vibrator. *V_p-p_* is deleted from the voltage of the HF rubber receptor, which is the built-in voltage generated by the ionized particles and molecules due to piezoelectricity, and the fluctuating deviation of every instrument. This deletion was also adopted in the subsequent figures. For the free nerve endings, the *V_p-p_* ratio in the horizontal vibration is larger than that in the case of vertical vibration because the U-rubber with receptor can be moved horizontally with relative ease, for example, during dynamic nodding movement, as shown in Figure 6b. However, for Pacinian corpuscles, the *V_p-p_* ratio in the vertical vibration is larger than not only horizontal vibration but also that in the case of free nerve endings. The cause of this discordance is guessed to be the difference between the structures of the receptor, which is multi-layered in one case and cylindrical in other. Incidentally, the typical property is that the effect of horizontal vibration on the equilibrium increases at a higher frequency in the free nerve endings.

In general, the equilibrium systems of artificial sensors designed using the proposed materials, which mimic semicircular canals, saccule, etc., are applicable as inertial sensors, accelerometers, and gyroscopes, which respond to dynamic inclination, such as acceleration, angular velocity, etc. [38,39,40,41,42]. In particular, the novel tilting sensor installed in a robot would be proposed for the state-of-the-art robotics. Although the results of the present study show static response to inclination along a single direction, the possibility of the dynamic inclination along all directions can be analyzed by expanding the present results. Furthermore, the demonstrated style, which uses HF rubber in the present study, would help design a novel tilt sensor that is different from other proposed tilt sensors that do not mimic biological organs [43].

## 8. Acoustic Performance

### 8.1. Methods

We validated the acoustic characteristics of HF rubber sensor, which functions as an acoustic sensor, such as hair cells in the cochlea. First, we investigated the effect of horizontal or vertical vibration on the HF rubber receptor proposed herein over low frequency range using mechanical vibrator, as shown in Figure 9b. Next, we used another vibration apparatus for high frequency oscillation, as shown in Figure 9a. Finally, regarding the sensitivity to auditory sound, we measured the acoustic level through each receptor or fabricated sensor to an applied sound from a speaker, as shown in Figure 9a,e.

### 8.2. Results and Discussion

**Figure 14:**
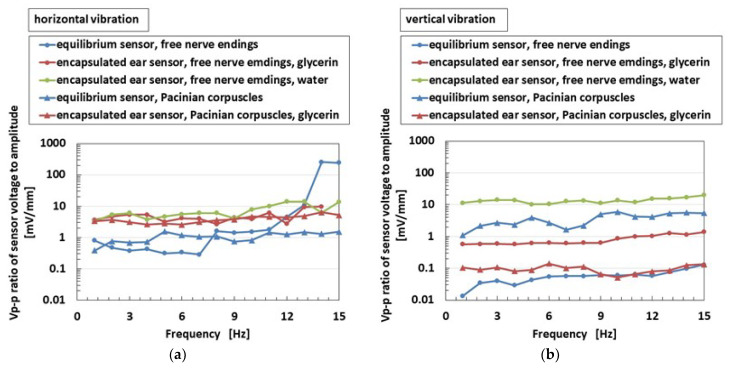
Experimental results of the vibrating auditory sensor by a mechanical vibrator when the vibration directions are compared: (**a**) horizontal vibration and (**b**) vertical vibration.

As for low frequency range oscillation, Figure 14 shows the comparison between equilibrium and ear sensors, which are two different kinds of HF rubber receptors, on the ratio of peak-to-peak voltage *V_p-p_* of the sensor to that of the 0.64 mm applied amplitude of the vibration. For the free nerve endings, the encapsulated ear sensor shown in Figure 4d has a *V_p-p_* ratio larger than that of the equilibrium sensor, except during lower frequency range, regardless of horizontal and vertical vibrations. For Pacinian corpuscles, the encapsulated ear sensor shown in Figure 4e exhibits a larger *V_p-p_* ratio for horizontal vibration, but a smaller *V_p-p_* ratio for vertical vibration than that of the equilibrium sensor. In this case, the variation is caused by difference in the receptor structure, which is multi-layered in one case and cylindrical in the other.

The human cochlea operates over a frequency band from around 20 Hz to 20 kHz [33]. In addition, to elucidate the auditory sensor by expanding the frequency range to 10,000 or 20,000 Hz from the low frequency range, as presented Figure 14, we used an encapsulated ear sensor that utilized another vibration apparatus, as shown in Figure 9a. This indicates our addressed high-frequency limit, considering the actual auditory circumstances of the human cochlea.

**Figure 15:**
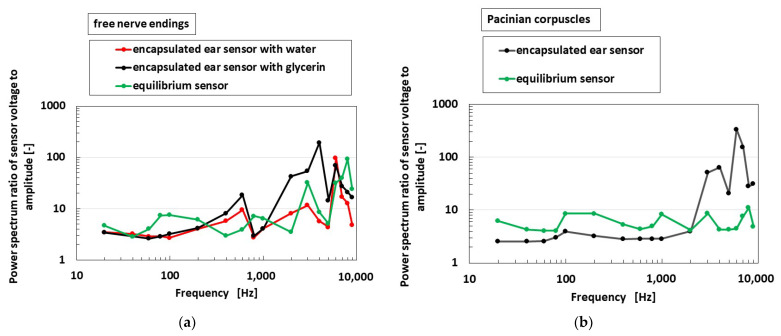
Experimental results of the vibrating auditory sensor with the vibration of a speaker when the receptor types are compared: (**a**) with free nerve endings and (**b**) with Pacinian corpuscles.

Figure 15 provides the ratio of the power spectrum between the voltage of the receptor and the applied amplitude of the vibration of the encapsulated ear sensor, which is shown in Figure 4d, by comparing to the equilibrium sensor similar to the comparison shown in previous figures in the last Section 7. The vibration is applied on a soft membrane of the sensor, as shown in the left side of Figure 9e, while using the experimental instrument, as shown in Figure 9a, spreading to the high frequency range. Beforehand, the displacement of Membrane 2 of the speaker, as shown in Figure 9a, was measured by touching a different load cell under the vibration and by having a frequency by the speaker, and the power spectrum of the vibration by load cell ** was simultaneously measured. In addition, we could obtain the numerical relation of the power spectrum between the displacement of Membrane 2 and the vibration by load cell ** as a calibration value. When using the calibration value, we can recognize the power spectrum of the displacement of Membrane 2 at the application of an electrical signal of the vibration from PC to the speaker. On the other hand, the power spectrum of the voltage from the sensor was measured using a voltmeter (PC710). Finally, the abscissa of the figure can be presented in the ratio of the power spectrum between the sensor voltage and the applied amplitude of the vibration of the encapsulated ear sensor or the speaker.

Here, a larger power spectrum ratio indicates a higher sensitivity of the receptor. The power spectrum is deleted from the voltage of the HF rubber receptor, which is the built-in voltage generated by the ionized particles and molecules through piezoelectricity, and the fluctuating deviation of every instrument, which is likewise adopted in the sequential figures. Moreover, the abscissa of the subsequent figures presents the same spectrum ratio.

**Figure 16:**
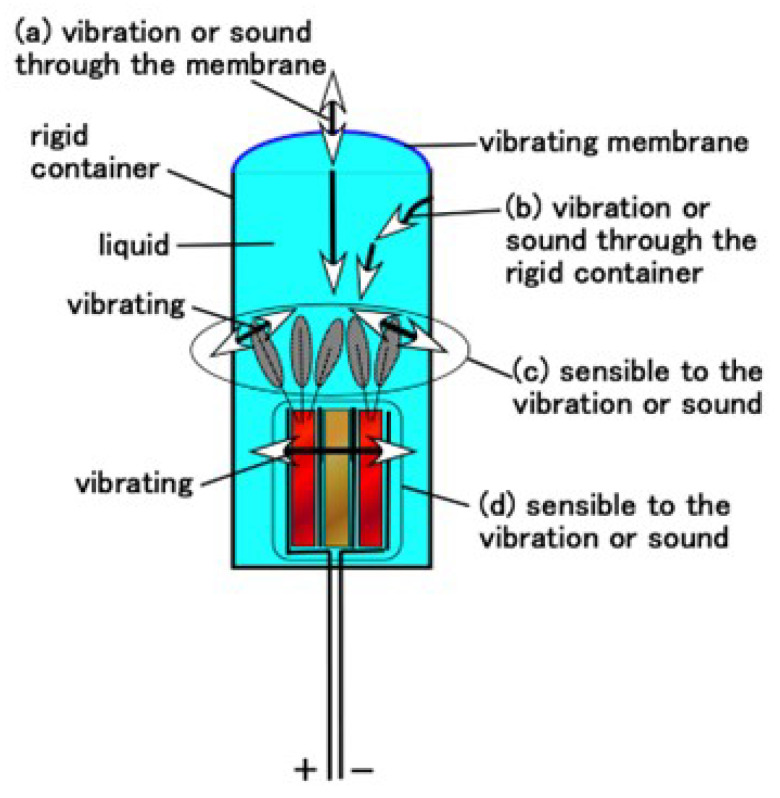
Schematic of physical model of acoustic performance by vibration of sound wave on auditory sensor mimicking the organ of Corti.

In the case of free nerve endings, it was confirmed that there is no typical quantitative distinction between the auditory and equilibrium sensors. However, for Pacinian corpuscles, their quantitative distinction is observed in low and high frequency ranges. The vibration of the sound wave is generated by the membrane and percolated through the liquid, as delineated by *a* and *b* in Figure 16, to reach on the hair and body, as delineated by *c* and *d* in Figure 16, respectively. The dielectricity of HF rubber 2, as shown Figure 4a,b, changes by the vibration of itself, as shown in *d*. In particular, the response of the hair is related to the vibration by the flow, as *c,* which corresponds to the flow sensing, such as other flow sensors proposed in the study of biomimetic soft materials [25,28,29]. The vibration of the hair as *c* affects the conductivity of HF rubber 4, as shown Figure 4a,b. Therefore, the present HF rubber receptor having hairs as *c* also demonstrates the effectiveness of flow sensor with utilizing hairs. As a result, from Figure 4a,b, the built-in voltage of the sensor changes.

The human cochlea involves other mammalian auditory systems and shows remarkable sensitivity to wide-ranged frequency. This sensitivity to the distinction of the wide-ranged frequency was realized by distributing the hair cells over a large cross-sectional area. The hair cells at the outer cochlea, which has a higher cross-sectional area than that of the innermost cochlea, is sensible to high frequency. In contrast, the hair cells at the innermost cochlea are sensible to low frequency. The investigation to demonstrate the auditory process has been conducted in previous studies [32,33,44].

**Figure 17:**
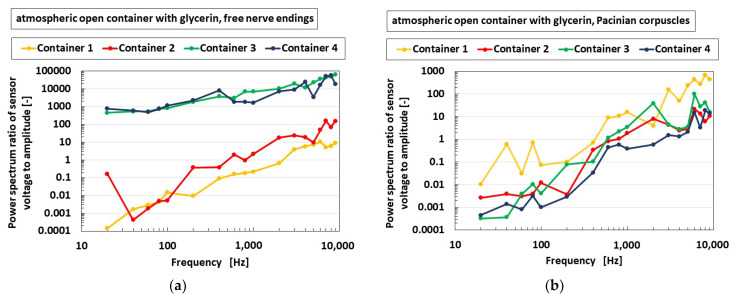
Experimental results of the vibrating auditory sensor with the vibration of a speaker when the container diameters are compared: (**a**) with free nerve endings and (**b**) with Pacinian corpuscles.

In the present study, we first attempted to elucidate the effect of the area of the container in which the HF rubber receptor is dipped on the auditory sensibility of the receptor. The top side of the container was not closed, which is similar to a human ear being open to the atmosphere, as shown in Figure 9c. As shown in Figure 17, when the diameter of the container was smaller, such as Containers 3 and 4, the free nerve endings were sensitive over a wide-ranged frequency. However, the Pacinian corpuscles were independent of the diameter of the container. As a result, the container requires small diameter in the case of free nerve endings, and the size of the diameter is irrelevant in the case of Pacinian corpuscles. Therefore, to obtain the auditory sensitivity by varying the diameter of the container, for example, to mimic the human cochlea, the free nerve endings serve as a better option of the diameter of the container.

**Figure 18:**
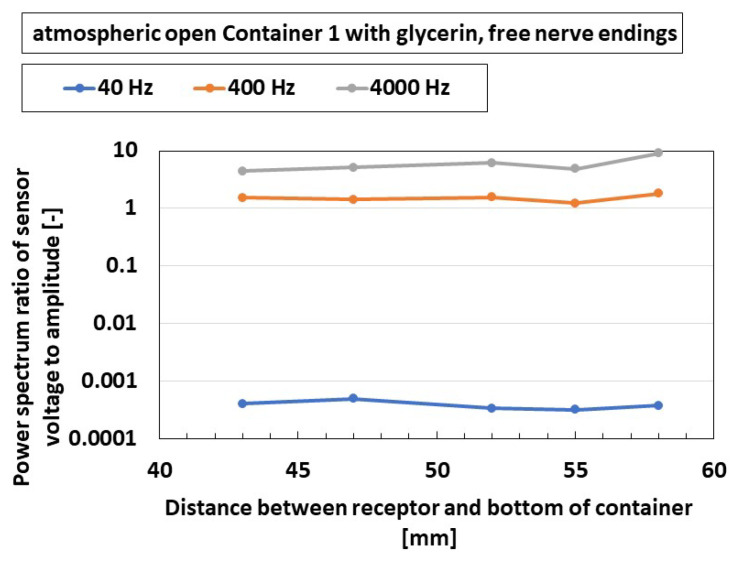
Experimental results of the vibrating auditory sensor with the vibration of a speaker when the frequencies are compared.

The next trial is to elucidate the effect of frequency and the position of the receptor on the auditory sensibility of the receptor. The experimental performance is the same as that of Figure 17, as shown in Figure 9c. The power spectrum ratio is independent on the position of the receptor over the present frequency range, as shown in Figure 18, which can be confirmed in any other cases involving any types of receptors, etc. The results depend on the range inner 50 mm height of the container; therefore, the receptor may be situated wherever they are, when the size of the encapsulated auditory sensor having less than 50 mm is produced.

**Figure 19:**
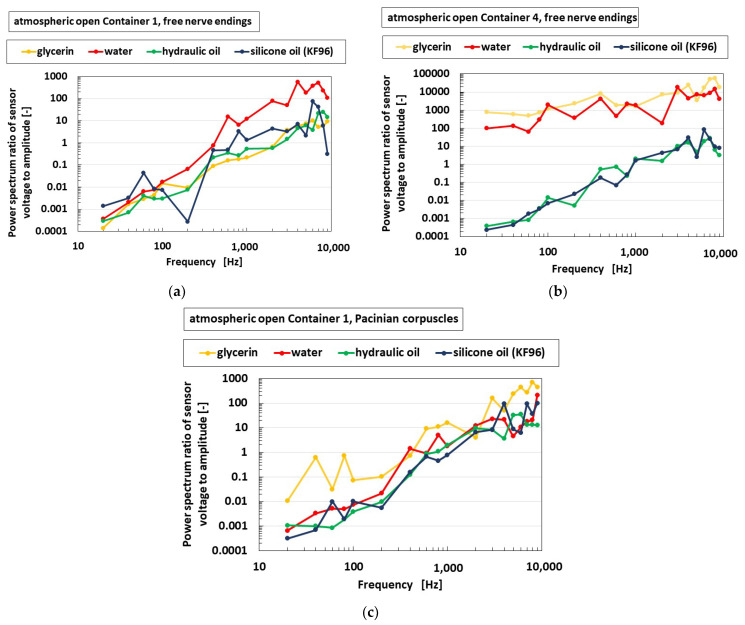
Experimental results of the vibrating auditory sensor with the vibration of a speaker when types of liquid are compared: (**a**) with free nerve endings in Container 1; (**b**) with free nerve endings in Container 4; (**c**) with Pacinian corpuscles.

Next, we studied the effect of the liquid, which is immersed in the container, on the auditory sensitivity of the receptor, as shown in Figure 19. The experimental performance is the same as that of Figure 17 and Figure 18, as shown in Figure 9c. The fluids present in the liquid are glycerin (1.261 × 10^3^ kg/m^3^ density, 1.412-Pas viscosity), water (0.998 × 10^3^ kg/m^3^ density, 9.799 × 10^−4^ Pas viscosity), hydraulic oil (0.866 × 10^3^ kg/m^3^ density, 3.456 × 10^−3^ Pas viscosity), and silicone oil (KF96, 0.969 × 10^3^ kg/m^3^ density, 6.627 × 10^−2^ Pas viscosity). The free nerve endings are independent of the kind of the liquid in the large diameter of the container. However, they are sensitive over a wide frequency range in the case of glycerin and water when the diameter is small. Nevertheless, these results do not imply that they depend on the container size. Furthermore, the results show that the Pacinian corpuscles are independent of the type of the liquid with a large diameter. As shown in the results of Figure 17 and Figure 19a,b, we considered glycerin or water and Container 4’s diameter size for producing the auditory encapsulated ear sensor, as shown in Figure 4d,e.

**Figure 20:**
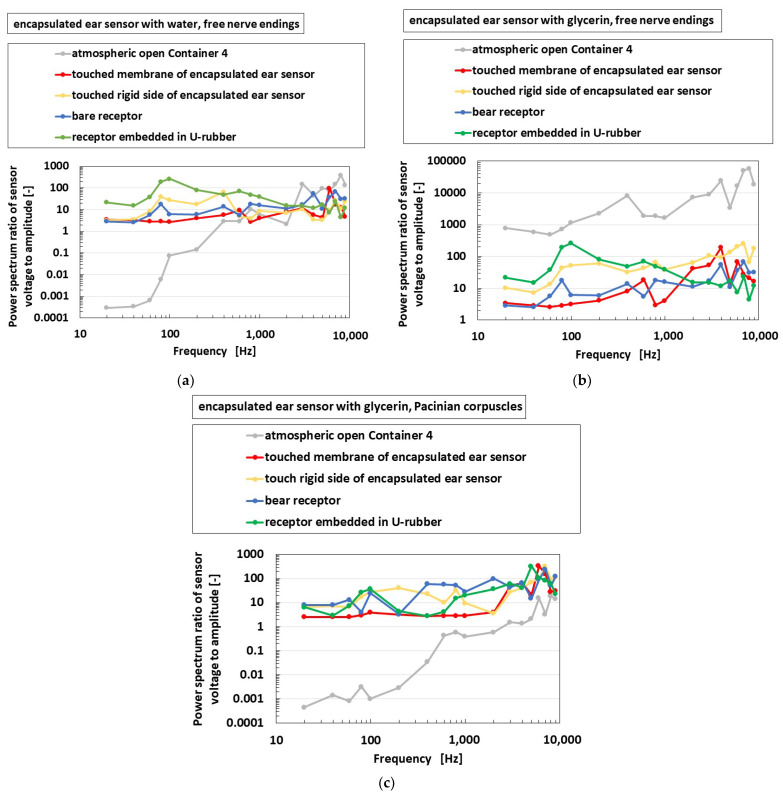
Experimental results of the vibrating auditory sensor with the vibration of a speaker when each receptor and sensor are compared: (**a**) with free nerve endings in water; (**b**) with free nerve endings in glycerin; (**c**) with Pacinian corpuscles.

Figure 20 shows the frequency property of the encapsulated auditory sensor shown in Figure 4d,e, which is labeled as “encapsulated ear sensor”, compared to the cases of the receptor dipped in a container, as shown in Figure 9c, which is labeled as “atmospheric open container”; the bare receptor directly in contact with the speaker without being dipped in any containers, as shown in Figure 9d, which is labeled as “bare receptor”; and the receptor embedded in U-rubber, as shown in Figure 4f, which is directly in contact with the speaker, which is labeled as “receptor embedded in U-rubber.” Considering the encapsulated auditory sensor, the vibration is applied on a soft membrane of the sensor, as shown in the left side of Figure 9e, and on a rigid lid side of the sensor, as shown in the right side of Figure 9e. In the case of free nerve endings, the osteophony, which refers to detecting the stimuli travelling across the bone as a rigid body, which involves the skull and inner ear, is more sensible than the case of the vibration through a liquid, such as cochlea at a lower frequency range, irrespective of whether water or glycerin were used. The sensitivity observed in the case of glycerin was larger than that in the case of water. As for water, the sensitivity of the bare receptor is smaller than that in other conditions, only at a lower frequency. However, for glycerin, the sensitivity of bare receptor is larger than that in other conditions over a wide frequency range. In the case of Pacinian corpuscles, the sensitivity of the bare receptor over a wide-rage frequency is lower than that in other conditions. Moreover, no quantitative difference was observed between these values.

Here, we investigate the relation between the equilibrium and acoustic sensing. Regrading equilibrium sensing, the receptor is covered by U-rubber. The change in voltage is induced by the deformation of the U-rubber, as shown in Figure 11, which creates the deformation of bilateral electric circuit of the receptor body as a condenser, as well as the deformation of the own receptor body, as shown as a-4 in Figure 4a. The deformation is created by the inclination, as shown in Figure 12. On the other hand, regarding acoustic sensing, the receptor is in bare state. The change in voltage is induced by the vibration of hair cells, as shown by c in Figure 16, as well as by the vibration of the receptor body as the own condenser, as shown by d in Figure 16. Their vibration is created by the sound wave. Thus, the mechanism of the generated voltage is different between the equilibrium and acoustic sensing. However, the phenomena that the hair cells and the receptor body are affected by the vibration or outer motion is the same.

**Figure 21:**
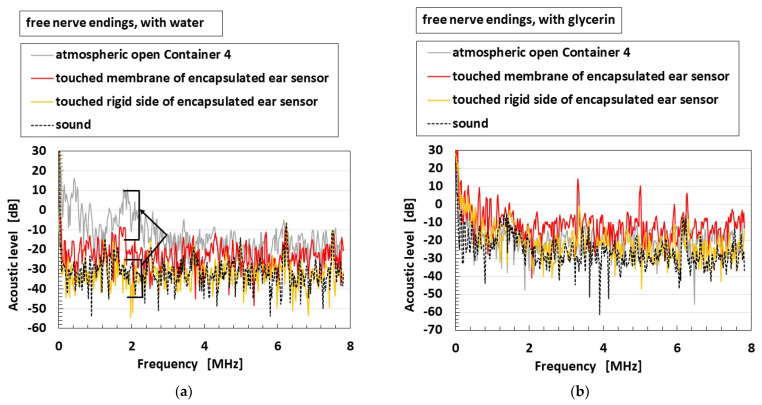

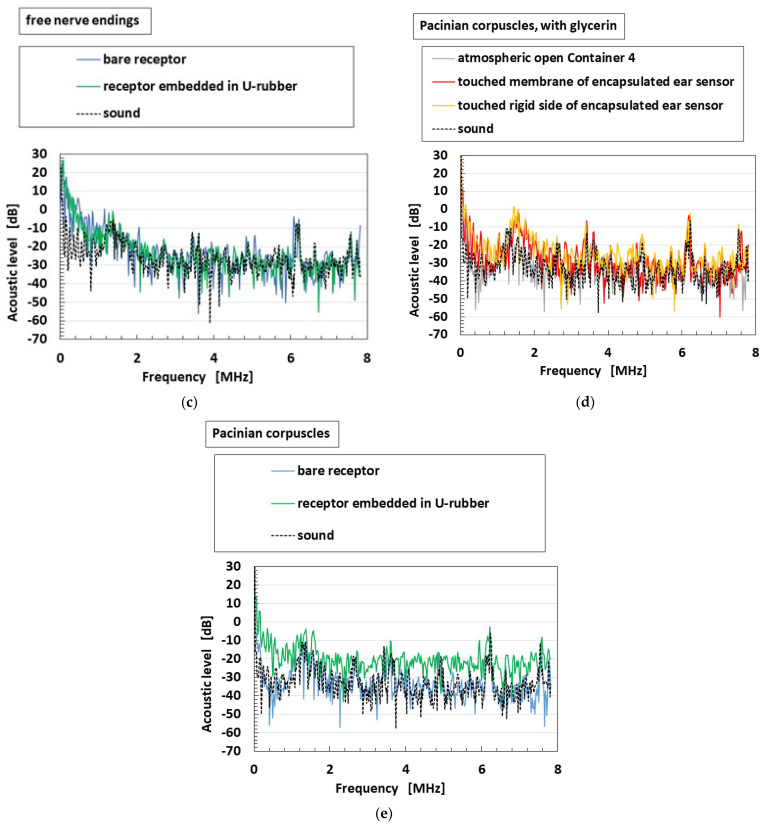
Experimental results of the vibrating auditory sensor on the sound level of a speaker when each receptor and sensor are compared: (**a**) with free nerve endings in water; (**b**) with free nerve endings in glycerin; (**c**) with free nerve endings; (**d**) with Pacinian corpuscles in glycerin; (**e**) with Pacinian corpuscles.

Finally, we elucidate the sensitivity to auditory sound. The measured voltage of the HF rubber receptor or sensor, which is built-in voltage generated by the ionized particles and molecules through piezoelectricity, directly creates a sound level that is sensitive to the applied sound wave. Therefore, by obtaining the voltage as an earphone, a headphone, or a speaker, we can hear the sound through the receptor or sensor. Figure 21 provides the acoustic level through each receptor or fabricated sensor to an applied sound from a speaker, as shown in Figure 9a,e. The experimental conditions, as shown in the figure, are the same as those in Figure 20. In the figure, the acoustic level of the applied sound is also shown. In the case of free nerve endings, particular for the encapsulated auditory sensor with water or glycerin, the acoustic level by the sensor becomes larger than that of the sound over every frequency, which is shown using a directed arrow in Figure 21a. However, for Pacinian corpuscles, the enhancement in acoustic level by the sensor is smaller than that of free nerve endings. In contrast, for the bare receptor and the receptor embedded in U-rubber, the enhancement in acoustic level in the case of free nerve endings is smaller than that of Pacinian corpuscles.

Incidentally, the equilibrium and acoustic sensors depend on the vibration or motion of hair cells or receptor body and of the U-rubber coating the receptor. Therefore, the conductivity of the liquid is independent of the equilibrium and acoustic properties. The viscosity of the liquid is one of factors affected on them.

**Figure 22:**
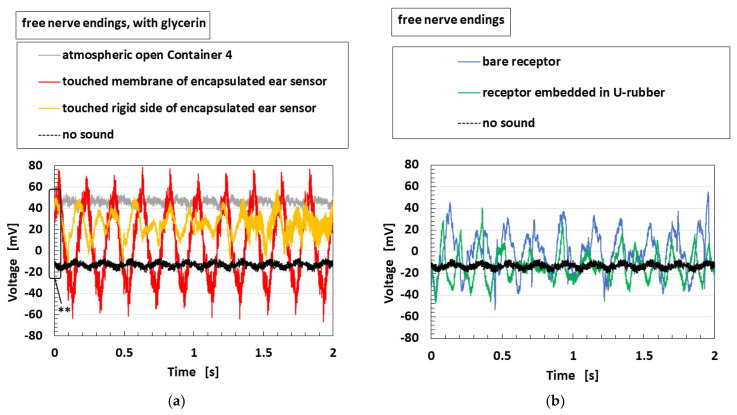
One example of the experimental results of the vibrating auditory sensor on the sound level of a speaker: (**a**) with free nerve endings in glycerin and (**b**) with free nerve endings.

Furthermore, we obtained the sound through the fabricated HF rubber receptor or sensor, such as an ordinary speaker with permanent magnet and coil. We showed one example of the change in the voltage of the fabricated sensor caused by the application of a sound (Figure 22), which is corresponding to the experimental conditions of Figure 21b,c. The initial voltage as delineated ** in Figure 22a means built-in voltage, which can easily change according to the experimental ambience, as is demonstrated in Figure 8b. It is relevant to the noise, and then we can reduce the noise if we manipulate some equalizers. Thus, because the voltage generated from the HF rubber sensor is affected by the sound, we can hear the sound by controlling the voltage with an equalizer.

From another perspective, the sound is amplified to be audible by the sensor in case of touching membrane of the auditory sensor, as well as in cases of touching the rigid side of the sensor and of receptor embedded in U-rubber. This means that the present sensor is effective for audibility to detect the sound by the vibration of the ear drum membrane, as well as by the oscillation of bone and muscles.

In conclusion, we can propose a novel speaker made of rubber without using a permanent magnet and a coil.

## 9. Conclusions

We addressed the establishment of state-of-the-art morphological production of an artificial intelligent soft-material for use in robotics. Then, we discussed its equilibrium and auditory sensing by utilizing our previously proposed technique of electrolytic polymerization on rubber. The electrolytic polymerization technique has the merit of less procedural steps for production. Furthermore, electrolytic polymerization on HF rubber enables not only the solidification of rubber but also the adhesion between metal wire as electrode and rubber. The produced prototypes are those of mimicked free nerve endings and Pacinian corpuscles, which are well-known receptors in the human skin. These devices have also sensory intelligence of tactile sensing to normal and shear forces and that of thermal sensing. This is due to the voltage of the HF rubber receptor, which is the built-in voltage generated by the ionized particles and molecules through piezoelectricity. Therefore, by integrating the HF rubber receptor and other materials, we proposed a novel rubber-made equilibrium and auditory sensors mimicking hair cells in the saccule and the cochlea at the vestibule of the human ear. The fabricated sensors can realize both equilibrium and auditory sensing. These characteristics and the specific performance of the equilibrium and auditory sensing vary based on the types of the HF rubber receptor, the types of liquid as lymph, size of container, etc. The optimal conditions of the fabrication can lead to a sufficiently high sensitivity. Regarding the morphological optimization of the receptor, further study of tactile, acoustic, etc., sensing the other receptors except for the nerve endings and Pacinian corpuscles, namely Meissner corpuscles, Krause and bulbs, Merkel cells, and Ruffini endings, are also needed. The typical morphological configuration of the receptor, including number and size of hair cells, arrangement, etc., that provides the optimization on the tactile, acoustic, etc., sensing would be presented. However, we obtained the effective design criteria for production of the rubber-made equilibrium and auditory sensors in the present study.

The present work demonstrated the viability of the rubber-made equilibrium and auditory sensor, which has the advantages provided by softness and elasticity, such as expansion and compression. This is different from the softness or deformation caused by bending, which has already been proposed in other studies by using artificial fabricate that is made using some thin-sheeted solid material, membrane, etc. In addition, HF rubber, as well as MCF rubber, has the variegated intelligence of self-powered electricity in the form of piezoelectricity, piezoresistivity, and photovoltaics. By reducing the size of the present equilibrium and auditory fabricate, they show increased engineering and biomechanical applicability in soft robotics. Therefore, our proposing auditory sensor has the novelty and possibility to be developed further for future applications, such as a novel speaker made of rubber without using a permanent magnet and a coil or a novel tilting sensor that responds to dynamic inclination, such as acceleration, angular velocity, etc., and is applicable to be installed in a robot. Therefore, the present equilibrium and auditory fabricate might also become a suitable aid for the elucidation of biological and mechanical peculiarity on the subcutaneous tissue and inner ear organ in the fields of dermatology and otolaryngology.

## Figures and Tables

**Table 1:** Ingredients of HF rubber for the fabrication of the receptor.

		HF Rubber 1 (For Hair Cell)	HF Rubber 2 (For Insulator as Condenser)	HF Rubber 3 (For Outer Cover)	HF Rubber 4 (For Adhesive)
**Ingredients**	Water	3 g	3 g	1 g	1 g
	Sodium tungstate (VI) dehydrate (Na_2_WO_4_·2H_2_O, Fujifilm Wako Chemical Co., Ltd., Osaka, Japan)	0.5 g	0.5 g	-	0.5 g
	TiO_2_ (Anataze type, Fujifilm Wako Chemical Co., Ltd., Osaka, Japan)	0.5 g	0.5 g	0.5 g	0.5 g
	HF	1 g	1 g	1 g	1 g
	NR-latex (Ulacol; Rejitex Co., Ltd., Atsugi, Japan)	3 g	3 g	3 g	3 g
	CR-latex (671A; Showa Denko Co., Ltd., Tokyo, Japan)	3 g	3 g	3 g	3 g
	Carbonyl Ni powder (No. 123, Yamaishi Co., Ltd., Noda, Japan)	3 g	3 g	3 g	3 g
Electrolytic polymerization conditions		20 V, 2.7 A	10 V, 2.7 A, 180 mT	6 V, 2.7 A, 180 mT	8 V, 2.7 A

## Data Availability

Not applicable.
